# Dynamic Association of ESCRT-II Proteins with ESCRT-I and ESCRT-III Complexes during Phagocytosis of *Entamoeba histolytica*

**DOI:** 10.3390/ijms24065267

**Published:** 2023-03-09

**Authors:** Mitzi Díaz-Hernández, Rosario Javier-Reyna, Diana Martínez-Valencia, Sarita Montaño, Esther Orozco

**Affiliations:** 1 Departamento de Infectómica y Patogénesis Molecular, Centro de Investigación y de Estudios Avanzados del IPN, México City 07360, Mexico; 2 Laboratorio de Modelado Molecular y Bioinformática, Facultad de Ciencias Químico-Biológicas, Universidad Autónoma de Sinaloa, Ciudad Universitaria s/n, Culiacán 80010, Mexico

**Keywords:** ESCRT machinery, ESCRT-II, *Entamoeba histolytica*, phagocytosis, virulence, EhADH, vesicular trafficking

## Abstract

By their active movement and voraux phagocytosis, the trophozoites of *Entamoeba histolytica* constitute an excellent system to investigate the dynamics of the Endosomal Sorting Complex Required for Transport (ESCRT) protein interactions through phagocytosis. Here, we studied the proteins forming the *E. histolytica* ESCRT-II complex and their relationship with other phagocytosis-involved molecules. Bioinformatics analysis predicted that EhVps22, EhVps25, and EhVps36 are *E. histolytica bona fide* orthologues of the ESCRT-II protein families. Recombinant proteins and specific antibodies revealed that ESCRT-II proteins interact with each other, with other ESCRT proteins, and phagocytosis-involved molecules, such as the adhesin (EhADH). Laser confocal microscopy, pull-down assays, and mass spectrometry analysis disclosed that during phagocytosis, ESCRT-II accompanies the red blood cells (RBCs) from their attachment to the trophozoites until their arrival to multivesicular bodies (MVBs), changing their interactive patterns according to the time and place of the process. Knocked-down trophozoites in the *Ehvps25* gene presented a 50% lower rate of phagocytosis than the controls and lower efficiency to adhere RBCs. In conclusion, ESCRT-II interacts with other molecules during prey contact and conduction throughout the phagocytic channel and trophozoites membranous system. ESCRT-II proteins are members of the protein chain during vesicle trafficking and are fundamental for the continuity and efficiency of phagocytosis.

## 1. Introduction

The endosomal sorting complex required for transport (ESCRT) is a conserved molecular machinery involved in cytokinesis, virus budding, endocytosis, and other cellular events that require membrane fusion, fission, scissoring, and repairing [1]. It has been studied in different organisms, from *Saccharomyces cerevisiae* [2] to *Homo sapiens* [3]. The proteins that constitute this machinery facilitate the movement of cargo from the limiting membranes through vesicles up to the MVBs [4,5], and oppositely, they participate in the movement of molecules from endosomes to other organelles, including the plasma membrane, to allow molecular trafficking and secretion [6]. These functions are performed mainly by the ESCRT-0, ESCRT-I, ESCRT-II, and ESCRT-III complexes, and the accessory proteins, Alix (a Bro-1 domain protein), which recognizes cargo, and Vps4 ATPase [7] that releases and recycles the ESCRT-III proteins after they have generated intraluminal vesicles (ILVs) in MVBs [8].

The ESCRT-II complex is a Y-shaped heterotetramer (2:1:1), composed of two Vps25 molecules in the arms, whereas Vps22 and Vps36 form the stalk. Vps25 possesses two PPXY sequences that bind to Winged Helix (WH-WH) motifs in Vps22 and Vps36. On the other hand, Vps36 has a Gram-like ubiquitin (GLUE) domain that connects Vps28, (ESCRT-I) [9,10], while Vps25 binds to Vps20 (ESCRT-III), forming a hypothetical bridge among ESCRT-I and ESCRT-III complexes. The Vps25 and Vps20 binding generate membrane curvature-sensing complexes [11], then Vps32 (ESCRT-III) interacts with Vps20 and sequentially assembles Vps24 and Vps2 [6]. This event permits the closure of the negatively deformed membranes containing the cargo [6]. In addition, Alix contacts Vps23 (ESCRT-I) and Vps32 (ESCRT-III) proteins contributing to the cargo progression during the whole process [12], it also promotes lysobiphosphatidic acid (LBPA) enrichment at the sites of ILVs formation [13]. The ESCRT machinery is involved in phagocytosis and virulence mechanisms of certain parasites [14]; thus, the detailed study of ESCRT molecules will help to understand these processes better and develop strategies to defeat parasitic infections.

The trophozoites of *Entamoeba histolytica*, the protozoan causative of human amoebiasis, present active phagocytosis and movement with extensive vesicle formation, in which the ESCRT machinery is deeply involved [15,16]. Earlier, 15 ESCRT genes were identified in the parasite [17,18], evidencing that *E. histolytica* possesses the conserved ESCRT machinery. Later, we studied the role of this molecular machinery in trophozoites. *E. histolytica* possesses the ancient ESCRT-0, ESCRT-I, ESCRT-II, and ESCRT-III complexes [18], which are relatively well-conserved. However, although *Ehhse1* and *Ehvps27 E. histolytica* genes (ESCRT-0) are transcribed [18], they lack Vps-27, Hrs and STAM domain (VHS) and Ubiquitin Interacting Motif (UIM) functional domains, and their secondary and tertiary protein structures exhibit poor homology with their putative orthologues [19]. Other proteins, including Tom-1, might carry out the ESCRT-0 tasks, as described for *Dictyostelium discoideum* [20]. In contrast, we have experimentally proved that the EhVps23 (ESCRT-I), EhVps2, EhVps20, EhVps24, and EhVps32 (ESCRT-III) proteins participate in phagocytosis, migration, and virulence of the parasite [14,15]. The knock-down of some of these genes affects the capability of the trophozoites to move and ingest RBCs [16,21]. The ESCRT-III complex was reconstructed using recombinant proteins and giant unilamellar vesicles (GUVs) to, in vitro, assign a functional role to its proteins during the ILVs formation in MVBs [16]. *E. histolytica* possesses the EhADH adhesin (an ALIX family protein) an accessory protein of the ESCRT machinery. During phagocytosis, EhADH contacts the prey, functioning as an adhesin. Then, it interacts with EhVps32 and EhVps23, reinforcing the bridge among the complexes [21,22], being present from the attachment and the capture of the prey up to its arrival to MVBs [23,24]. EhVps4-ATPasa, another accessory protein of the ESCRT machinery, is also present in *E. histolytica*, and as in other systems, it disassembles the ESCRT-III complex, liberating the proteins to be recycled.

However, the three proteins of the ESCRT-II that constitute the bridge between ESCRT-I and ESCRT-III complexes have not been studied yet in *E. histolytica*. To approach a panoramic understanding of the role of the ESCRT machinery in phagocytosis, in this study, we cloned *Ehvps22*, *Ehvps25*, and *Ehvps36* genes and characterized their protein structure and function, analyzing the putative interactions among them and with other proteins. We also studied the role of the ESCRT-II complex in phagocytosis using knocked-down trophozoites in one of its genes.

## 2. Results

### 2.1. EhVps22, EhVps25, and EhVps36 Possess Functional WH-WH Domains

EhVps22, EhVps25, and EhVps36 full-length amino acid sequences obtained from the AmoebaDB were aligned with the ones corresponding to their *H. sapiens* and *S. cerevisiae* orthologues. Analysis demonstrated that, like other *E. histolytica* proteins, they conserve the FY, IE, NGG, SVP, and YW amino acids that conform to the WH-WH domain (Figures S1–S4) involved in DNA- and RNA-binding, as well as to certain proteins [25]. Vps36 contains two Npl4 zinc finger motifs at the N-terminal region of the WH domains [5] that are called zinc finger (NFZ) in yeast and GLUE in humans, but it does not have the GLUE domain that connects to the Phosphatidylinositol-3,4,5-triphosphate (PIP3) lipid that supports the sorting of ubiquitinated cargo in other systems (Figure S4), suggesting that other proteins might participate in this event.

**Figure 1:**
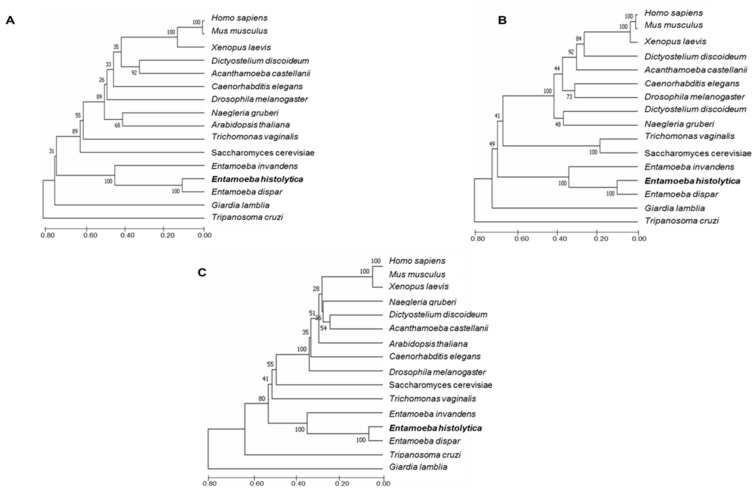
Phylogenetic trees of EhVps22, EhVps25, and EhVps36. (**A**–**C**) ESCRT-II proteins could come from a common ancestor with other organisms. The position of *E. histolytica* ESCRT-II proteins is in bold. Left numbers: confidence percentages of the tree topology by bootstrap analysis of 1000 replicates.

Interestingly, in the phylogenetic trees, the three *E. histolytica* ESCRT-II components appeared in a similar position, between *Entamoeba dispar* and *Entamoeba invadens*, close to *Giardia lamblia* and *Trypanosoma cruzi*, and far from *H. sapiens* (Figure 1A–C). This could suggest that *E. histolytica* ESCRT-II proteins could come from a common ancestor. However, more studies are necessary to obtain further evidence for this hypothesis.

### 2.2. The Three-Dimensional Structures of EhVps22, EhVps25, and EhVps36 Proteins Present high HOMOLOGY with Their Orthologues

Pivotal information about the protein’s membership in each family is provided by quantitative comparison of molecular structures. Furthermore, the protein structure and dynamics help predict putative associations with other molecules. Here, to obtain further evidence that EhVps22, EhVps25, and EhVps36 are bona fide orthologues of their respective families, we obtained the three-dimensional (3D) structures of the ESCRT-II proteins and analyzed them by pairwise comparison with their human and yeast orthologues. The best 3D models were submitted to molecular dynamics simulation (MDS). We calculated the root-mean square deviation (RMSD) to measure the similarity between superimposed atomic coordinates during the time-lapse and ascertain when the protein reaches its stability. In addition, we also estimated the root-mean square fluctuations (RMSF) to measure the displacement of the Cα and the radius of gyration (Rg) to determine the compactness of the structure [26].

**Figure 2:**
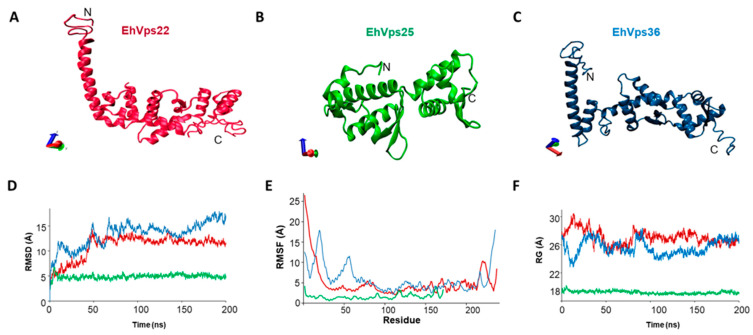
3D structures of EhVps22, EhVps25, and EhVps36 and MD simulation. (**A**–**C**) 3D model of EhVps22, EhVps25, and EhVps36. (**D**) RMSD. (**E**) RMSF. (**F**) Rg: Radius.

The EhVps22 3D structure contains nine α-helices and three β-sheets that form the two WH domains, as described for the *S. cerevisiae* and *H. sapiens* orthologues [10]. The structural alignment of this model exhibited an RMSD of 1.35 and 2.88 Å with human and yeast crystal orthologues, respectively (Figure S5). After RMDS, EhVps22 reached its stability at 60 ns (Figure 2D), whereas the RMSF exhibited three areas of a major movement in the protein, one at M1-F23 with a fluctuation from 20 Å, probably due to the lack of secondary structure at the N-terminus of the protein (Figure 2E). The second one was found at W214-A228 with fluctuations of 10 Å, and the third one at F237 amino acid with fluctuations of 8 Å (Figure 2E), both located at the C-terminus, which is composed of coiled structures. The Rg showed protein expansion in the first 15 ns and compactness from 16 to 60 ns of the trajectory, reaching stability after 80 ns of MDS (Figure 2F).

EhVps25 presents six α-helices and eight β-strands; meanwhile, the WH domains appeared formed by five alpha helices (Figure 2B) as in its orthologues [11]. The structural alignment of this model exhibited an RMSD of 0.88 and 2.76 Å with human and yeast crystals orthologues, respectively (Figure S5). EhVps25 reached its stability at the beginning of RMDS at 20 ns (Figure 2D), whereas the RMSF exhibited five areas of major movement at N89-S91 with 3 Å, G115-G119, with fluctuations of 3.5 Å, N129-E132, and Q160-E164 and another in K171 amino acids with fluctuations of 3 Å, 4 Å, and 3.5 Å, respectively (Figure 2E). The Rg indicated protein expansion in the first 10 ns of the MDS, and then, the system remained without changes after 190 ns of the trajectory, which coincides with the RMSD values (Figure 2F).

The EhVps36 model appeared to be formed by eight α-helices and five β-sheets (Figure 2C). The WH domains, except for the last β sheet, is located at the C terminus. EhVps36 reached stability after 170 ns of simulation (Figure 2D). The RMSF exhibited three regions of fluctuation located at M1-S28, D45-E60, and T228-K235 with 18 Å, 12 Å, and 18 Å, respectively (Figure 2E). The Rg calculations indicated that EhVps36 is a highly dynamic protein that expands and compacts during the trajectory (Figure 2F). The EhVps36 structure presented an RMSD value of 1.69 and 2.7 Å, respectively, compared with yeast and human orthologues (Figure S5). All these data strongly suggest that EhVps22, EhVps25, and EhVps36 are bona fide members of the ESCRT-II families.

### 2.3. WH-WH Domains Key Regions for Interaction between EhVps22, EhVps25, and EhVps36 by In Silico Analyses

**Figure 3:**
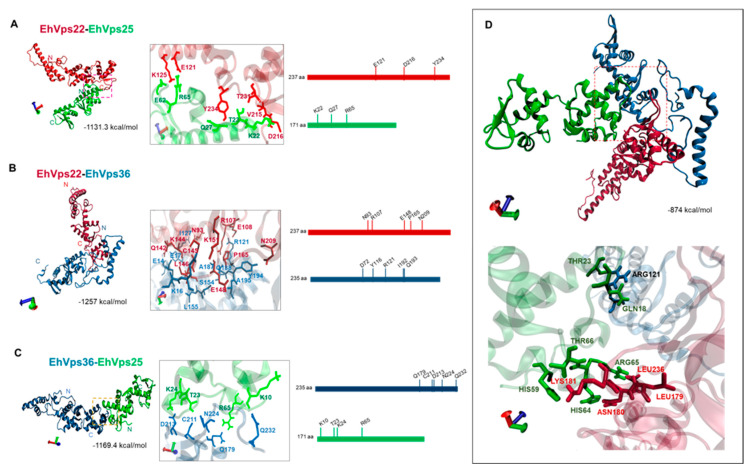
Molecular docking between EhVps22, EhVps25, and EhVps36 proteins. (**A**) Docking of EhVps22 with EhVps25. (**B**) Docking of EhVps22 andEhVps36. (**C**) Docking between EhVps36 with EhVps25. Right panels: zoom of the squared regions in (**A**–**C**) involved in binding. The next panel is the zoom; linear representation of the amino acid position involved in the association between the proteins. (**D**) In silico analysis of the three interacted proteins as a single complex. Zoom of the squared regions in (**D**).

After obtaining the average conformers of stable proteins, we explored by molecular docking the contact among EhVps22, EhVps25, and EhVps36, detecting that the amino acids involved belong to regions located in the WH domains of each protein. EhVps22 binds to EhVps25 with -1131.3 kcal/mol of free-binding energy (Figure 3A), through the residues GLU121, LYS125, VAL215, ASP216, THR231, and TYR234; most of them are located in the second peak of fluctuation, where the amino acids lack secondary structure (Figure 3A,C). In contrast, ARG65, GLU62, LYS22, THR23, and GLN27 amino acids participate in EhVps25 (Figure 3A). EhVps22 interacts with EhVps36 with an energy of -1257 kcal/mol (Figure 3B) through ASN36, LYS39, LYS79, TYR81, ASN83, ILE84, ASN93, GLU101, ARG107, GLU108, GLN142, LYS144, CYS145, LEU146, GLU148, LYS151, PRO165, and ASN209 amino acids, whereas TYR40, LEU71, ASP72, ASN29, THR36, LYS10, TYR116, ILE127, ARG121, GLN188, GLN193, ALA187, ILE192, GLU14, GLU17, LYS16, LEU155, SER154, TYR194, and ALA195 amino acids participate in EhVps36 (Figure 3B). On the other hand, the molecular docking between EhVps36 and EhVps25 revealed that these proteins could interact with an energy of -1169.4 kcal/mol (Figure 3C). In the model, the interaction was carried out in EhVps25 through ARG65, THR23, LYS10 and LYS24 amino acids, while in EhVps36, theGLN179, CYS211, ASN224, GLN232, and ASP213 amino acids participated (Figure 3C). In addition, we performed the docking of the three proteins. The analysis indicated that the expected protein conformation changes to adapt to the novel interactions. Some of the same amino acids involved in the predicted interaction with a single protein could be involved (Figure 3D). These analyses predicted that EhVps22, EhVps25, and EhVps36 could form the ESCRT-II complex by interacting among them.

### 2.4. The ESCRT-II Proteins Are Located in the Cytoplasm of the Trophozoites, and Co-Localization Was Observed in Vesicles and Near the Plasma Membrane

**Figure 4:**
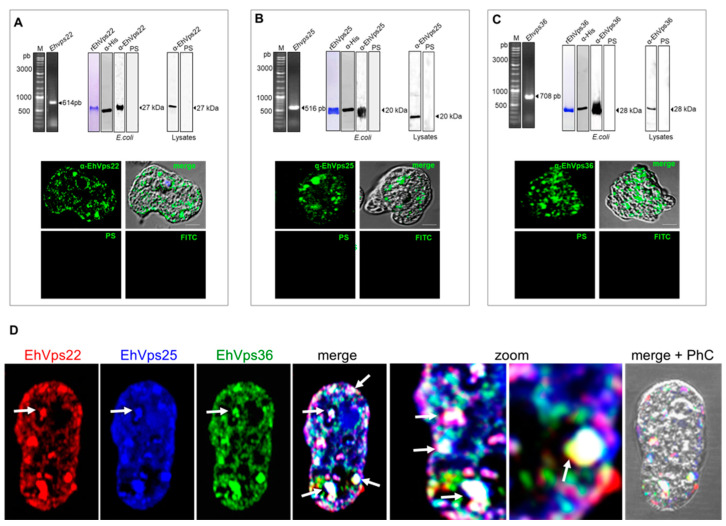
Cellular localization of ESCRT-II proteins in *E. histolytica* trophozoites. (**A**–**C**). Cloning, expression, and amplification of the respective genes. (**A**) Detection of EhVps22, (**B**) EhVps25, and (**C**) EhVps36 proteins in bacterial and amoebic lysates. PS: pre-immune serum. The lower images show the cellular localization of EhVps22, EhVps25, and EhVps36 proteins in the trophozoites. Negative controls: PS and secondary antibody (FITC). (**D**) Images of trophozoites under basal conditions showing the co-localization sites of EhVps22 (red), EhVps25 (blue), and EhVps36 (green) proteins. PhC: phase contrast images. Arrows and zoom indicate co-localization sites between proteins. Scale bar = 10 µm.

The cellular location of a protein also gives clues about its function; therefore, we scrutinized the proteins inside the cell, searching for putative interactions among them and with other molecules. We cloned the genes to obtain the histidine-tagged recombinant proteins (Figure 4A–C) and generated specific polyclonal antibodies for each protein in distinct animal species. In western blot assays, the antibodies recognized specific bands of 27, 20, and 28 kDa, corresponding to the EhVps22, EhVps25, and EhVps36 expected molecular weights, respectively, calculated according to their amino acid sequence (Figure 4A–C). In the basal state, the laser confocal microscopy images revealed the proteins dispersed in the cytoplasm of the trophozoites, surrounding some vesicles, in pseudopodia, and as aggregates close to the internal plasma membrane (Figure 4A–C). Co-localization among them was observed in specific vesicles and near the plasma membrane (Figure 4D).

### 2.5. The ESCRT-II Proteins Change Their Location during Phagocytosis

**Figure 5:**
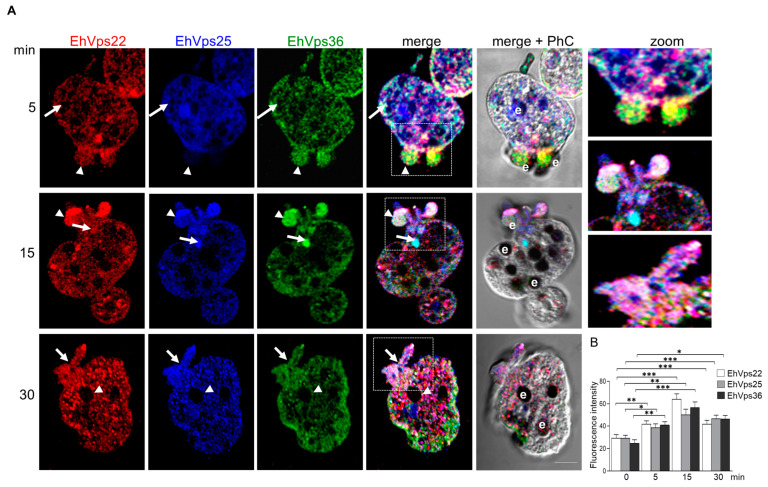
Cellular localization of the ESCRT-II proteins during phagocytosis. (**A**) Trophozoites in basal condition treated with α-EhVps22 (red), α-EhVps25 (blue), and α-EhVps36 (green) antibodies. Arrows indicate the co-localization sites of the three proteins. Arrowheads indicate the co-localization sites on or surrounding the erythrocytes. e: erythrocyte. Bar = 10 μm. Zoom: magnification of the regions marked with white squares in the zoom areas. PhC: phase contrast images. (**B**) Fluorescence intensity was measured by pixels in 30 cells in basal condition and after phagocytosis. (*) *p* < 0.05, (**) *p* < 0.01 (***) *p* < 0.001.

EhVps23 (ESCRT-I) and the four proteins of the ESCRT-III complex are deeply involved in phagocytosis [14,21]. Here, we investigated whether the ESCRT-II was also a clue part of this event. We carried out kinetics of phagocytosis from 0 to 30 min, and then protein location was analyzed by confocal microscopy using the specific antibodies for each protein. At 5 min, we detected the proteins around certain RBCs attached to the trophozoites plasma membrane, apparently in the process of ingestion (Figure 5). The proteins were also found in the RBCs containing phagosomes, near to the endosomes and in the phagocytic cups. Intriguingly, not all the bound RBCs were marked by the antibodies. We hypothesize that this is because they were attached to the trophozoites at different times. The image in Figure 5A at 5 min shows a pair of RBCs stained mainly by EhVps36 and EhVps22, but in other images, the three proteins appeared to be co-localizing (Figure 5A, 15 min). After 30 min, the proteins emerged together or separated, as we found them in basal conditions. Differences in fluorescence intensity were also observed in the images (Figure 5B). The results of these experiments suggest that ESCRT proteins are rapidly re-localizing to different cellular compartments during phagocytosis.

### 2.6. The ESCRT-II Proteins BIND ESCRT-I and ESCRT-III Complexes, and Their Interaction Increases during Phagocytosis

**Figure 6:**
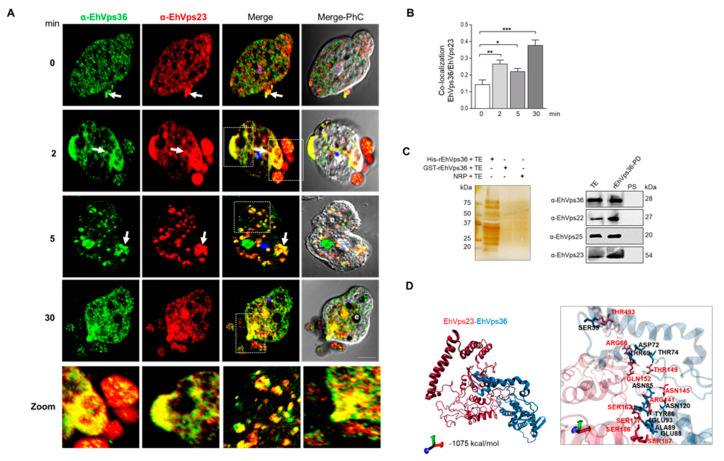
Cellular co-localization of EhVps36 (ESCRT-II) and EhVps23 (ESCRT-I) during phagocytosis. (**A**) Confocal images of trophozoites treated with α-EhVps36 (green) and α-EhVps23 (red) antibodies. The nucleus was stained with DAPI (blue). PhC: phase contrast images. Arrows: co-localization of the proteins. Zoom: magnification of the regions marked with white squares. Bar = 10 µm. (**B**) Pearson’s coefficient of co-localization of EhVps36 and EhVps23 (*) *p* < 0.05, (**) *p* < 0.01 (***) *p* < 0.001. (**C**) Left panel: silver-stained gel of the samples obtained from the pull-down assay and negative controls. TE: total extracts; NRP: non-recombinant protein. Right panel: western blot assay of pull-down assays using α-EhVps36 for precipitation and α-EhVps22, α-EhVps25, and α-EhVps23 antibodies to detect the associated proteins. rEhVps36-PD: pull-down sample PS: pre-immune serum. Numbers to the right: molecular weights. (**D**) Molecular docking between EhVps36 and EhVps23. The inset shows a magnification of the predicted amino acids involved in protein–protein interaction.

**Figure 7:**
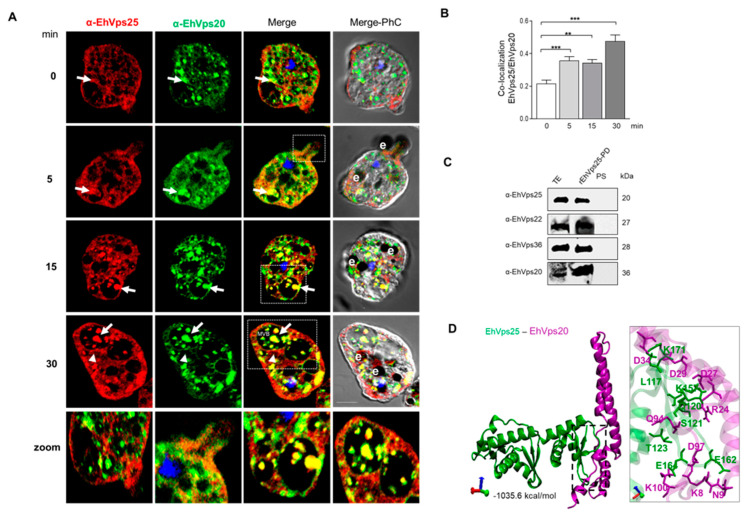
Co-localization of EhVps25 (ESCRT-II) and EhVps20 (ESCRT-III) during phagocytosis. (**A**) Confocal images of trophozoites treated with α-EhVps25 (red) and α-EhVps20 (green) antibodies. The nucleus was stained with DAPI (blue). PhC: phase contrast images. Arrows: protein co-localization. Zoom: magnification of the region marked with white squares. Bar = 10 μm. (**B**) Pearson’s coefficient of EhVps25 and EhVps20 co-localization (**) *p* < 0.01, (***) *p* < 0.001. (**C**) Western blot assay of pull-down assays using α-EhVps25, α-EhVps22, α-EhVps36 and α-EhVps20 antibodies and extracts from trophozoites in basal conditions. TE: total extracts; rEhVps25-PD: pull-down sample PS: pre-immune serum. Numbers at the right: molecular weights. (**D**) Molecular docking between EhVps25 and EhVps20. The box shows a magnification of the putative interacting amino acids.

We and others have proven that several proteins of ESCRT-III and ESCRT-I complex attach and detach among them and in a dynamic way [24,27,28,29]; however, we do not know yet whether at a given moment, the complexes appear fully assembled. In a hypothetical temporary complex, very difficult to detect by these techniques, ESCRT-II must be the link between ESCRT-I and ESCRT-III, as many authors have suggested [9,10,11]. Thus, we analyzed the interaction between the ESCRT-II and ESCRT-I and the binding between ESCRT-II and ESCRT-III by confocal microscopy and pull-down experiments, followed by the identification of certain proteins by western blot assays and mass spectrometry analysis. For these experiments, we used the specific antibodies α- EhVps36 (ESCRT-II) and α-EhVps23 (ESCRT-I), or α-EhVps25 (ESCRT-II) and α-EhVps20 (ESCRT-III) (Figure 6 and Figure 7). Confocal images showed that after 2 min of phagocytosis, EhVps36 and EhVps23 co-localized on the adhered RBCs in phagosomes and in vacuoles close to the erythrocytes (Figure 6A). At 5 min, fluorescence still appeared in the cytoplasm, associated with ingested erythrocytes, and at 30 min, we found them in RBCs close to the trophozoites. Probably, some RBCs were trapped by a certain trophozoite thin membrane that was not revealed here by confocal microscopy. The label was also found in the phagocytic channel. Although we observed co-localization of the ESCRT-II proteins on the RBCs and in the channels, we also detected the fluorescent antibodies as separated. Remarkably, as in other experiments, the co-localization of both antibodies changed according to the time of phagocytosis, evidencing the dynamics of the event. Similarly, images obtained using α-EhVps25 (ESCRT-II) and α-EhVps20 (ESCRT-III) antibodies supported the co-localization of the two ESCRT complexes in the phagocytic channels, in phagosomes, in ILVs inside MVBs, and vacuoles close to the RBCs (Figure 7A), that is, through the whole phagocytosis process. These results demonstrate that ESCRT-I binds, directly or indirectly, to ESCRT-II through the EhVps36 (ESCRT-II) and EhVps23 (ESCRT-I) proteins and to ESCRT-III by EhVps25 (ESCRT-II) and EhVps20 (ESCRT-III). However, these results do not discard the participation of other proteins in the interaction.

To obtain more data to support the nature of the interactions between the ESCRT complex, we performed a pull-down analysis using the recombinant EhVps25 and EhVps36 proteins, independently incubated with amebic extracts. Subsequently, the samples obtained from the pull-down were analyzed by western blot.

As expected, when we used recombinant EhVps36 to carry out pull-down in the immunoprecipitate, we found the EhVps22, EhVps25, and EhVps23 proteins (Figure 6C). On the other hand, recombinant EhVps25 detected EhVps22, EhVps36, and EhVp20 proteins in the mixture (Figure 7C). These results confirmed the molecular docking predictions and the laser confocal microscopy studies, strongly suggesting that all these proteins are in permanent movement and interact directly or indirectly, which is more evident during phagocytosis.

We performed molecular docking analysis to predict the amino acids involved in these bindings. The interaction between EhVps36 and EhVps23 (Figure 6D) was performed by −1075 kcal/mol free binding energy, further supporting the confocal microscopy results. This binding site appeared to be formed by two salt bridges and 11 hydrogen bonds (Figure 6D). The residues from EhVps23 involved were ARG66, ARG141, ASN145, THR149, GLN152, SER162, SER177, SER186, SER187, and THR493; for EhVps36, they were ASP72, GLU93, GLY124, ASN120, THR74, THR69, TYR86, GLU93, ALA89, GLU88, and SER55. Meanwhile, the free-binding energy for the interaction between EhVps25 and EhVps20 (Figure 7D) was -1035.6 kcal/mol, mediated by five salt bridges and six hydrogen bonds (Figure 7D). For the EhVps25–EhVps20 interaction, the involved amino acids were GLU164, GLU162, THR123, SER121, GLN120, LYS157, LYS117, and LYS171 in EhVps25 and LYS8, ASP97, LYS100, ASN9, GLN94, ARG24, ASP27, ASP34, and ASP29 in EhVps20.

### 2.7. Mass Spectrometry Analysis Reveals the Presence of Proteins Involved in Phagocytosis

The samples obtained from pull-down experiments were submitted to mass spectrometry analysis, and the proteins that exhibited more than 95% reliability are in Table 1. Interestingly, after the depuration, most of the proteins that appeared with the highest reliability percentage are related to phagocytosis and virulence processes. The pull-down samples obtained using the rEhVps22 protein revealed that in addition to the ESCRT-II proteins, were detected EhVps29, a protein of the retromer complex whose function is protein recycling and is linked to the ESCRT machinery [30,31,32], the EhRabB protein [33,34,35], ARP 2/3, tubulin, actin, reticulin, Gal/GalNac [36], HSP70, and hexokinase 1 [37] (Table 1), most of them experimentally associated to phagocytosis.

On the other hand, the mass spectrometry analysis revealed in pull-down samples obtained with rEhVps25, the presence of EhVps22 and EhVps36 (ESCRT-II), and EhVps20 and EhVps32 (ESCRT-III), as well as EhRabB [35], ARP 2/3 [38] and grainin1 [39] (Table 1). These experiments strongly suggest that the EhVps36 protein associates, directly or indirectly, with EhVps22 and EhVps25, EhVps23 of the ESCRT-I, and EhVps32 of ESCRT-III (Table 1). Other proteins identified in this analysis were calmodulin [40], grainin, clathrin [41], and EhCBP1 [39]. These results strongly suggest that in *E. histolytica*, ESCRT-II binds to ESCRT-I and ESCRT-III complexes and interacts directly or indirectly with other proteins involved in phagocytosis, supporting the interplay of molecules participating in the event and the dynamism of the temporary binding among the ESCRT-II complex proteins, and with the ESCRT-I and ESCRT-III proteins to allow the continuity of the process.

**Table 1:** rEhVps25, and rEhVps36 with proteins involved in phagocytosis and cytoskeleton. Data were obtained by mass spectrometry analysis with a reliability percentage >95%. The database AmoebaDB (https://amoebadb.org/amoeba/app, accessed on November 2022) was used to provide the accession number of each protein.

Recombinant Protein	Access Number	Protein	Function	Reference
**EhVps22**	EHI_181240	Rab B GTPase	Phagocytosis	Javier-Reyna et al., 2019 [34]
	EHI_137860	EhVps25	Phagocytosis (ESCRT-II)	López-Reyes et al., 2010 [18]
	EHI_045320	EhVps36	Phagocytosis (ESCRT-II)	López-Reyes et al., 2010 [18]
	EHI_025270	EhVps29	Phagocytosis (Retromer complex)	-----------
	EHI_030820	ARP2/3 complex 20 kDa subunit	Phagocytic cups and cytoskeleton	Mrigya-Babuta et al., 2015 [38]
	EHI_182900	Actin	Cytoskeleton	Parimita Rath et al., 2020 [38]
	EHI_049920	Tubulin	Cytoskeleton (microtubule nucleation)	Gilchrist et al., 1999 [36]
	EHI_136160	Calreticulin putative	Development of amoebic liver abscesses	Enrique-González et al., 2011 [32]
	EHI_012270	Gal/GalNAc lectin heavy subunit	Adherense and cytolysis	Gilchrist et al., 1999 [36]
	EHI_052860	Heat shock protein 70	Amoebic pathogenicity by protection against oxidative and nitrosative stress	Gilchrist et al., 1999 [36]
	EHI_098290	Hexokinase 1	Flux control of glycolysis	Saucedo-Mendiola et al., 2014 [37]
**EhVps25**	EHI_181240	Rab B GTPase	Phagocytosis	Javier-Reyna et al., 2019 [34]
	EHI_131120	EhVps22	Phagocytosis (ESCRT-II)	López-Reyes et al., 2010 [18]
	EHI_045320	EhVps36	Phagocytosis (ESCRT-II)	López-Reyes et al., 2010 [18]
	EHI_066730	EhVps20	Phagocytosis (ESCRT-III)	Ávalos-Padilla et al., 2018 [22]
	EHI_169820	EhVps32	Phagocytosis (ESCRT-III)	Ávalos-Padilla et al., 2018 [22]
	EHI_030820	ARP2/3 complex 20 kDa subunit	Phagocytic cups and cytoskeleton	Mrigya-Babuta et al., 2015 [38]
	EHI_167300	Grainin 1	Vesicular maturation and exocytosis and Programmed cell death	Tovar et al., 2000 [41]
**EhVps36**	EHI_181240	Rab B GTPase	Phagocytosis	Javier-Reyna et al., 2019 [34]
	EHI_135460	EhVps23	Phagocytosis (ESCRT-I)	Galindo-Olea et al., 2021 [15]
	EHI_131120	EhVps22	Phagocytosis (ESCRT-II)	López-Reyes et al., 2010 [18]
	EHI_137860	EhVps25	Phagocytosis (ESCRT-II)	López-Reyes et al., 2010 [18]
	EHI_169820	EhVps32	Phagocytosis (ESCRT-III)	Ávalos-Padilla et al., 2018 [22]
	EHI_023500	Calmodulin, putative	Phagocytosis and actin dynamics	Rout et al., 2011 [40]
	EHI_120360	Grainin	Associated with reduced virulence	Tovar et al., 2000 [41]
	EHI_201510	Clathrin heavy chain, putative	Secretion	Rosalinda-Tovar et al., 2000 [41]
	EHI_120900	Calcium-binding protein 1 (EhCBP1)	Phagocytic cups and cytoskeleton dynamics	Nickel et al., 2000 [39]

### 2.8. The ESCRT-II Complex Is Involved in Phagocytosis and Adherence

**Figure 8:**
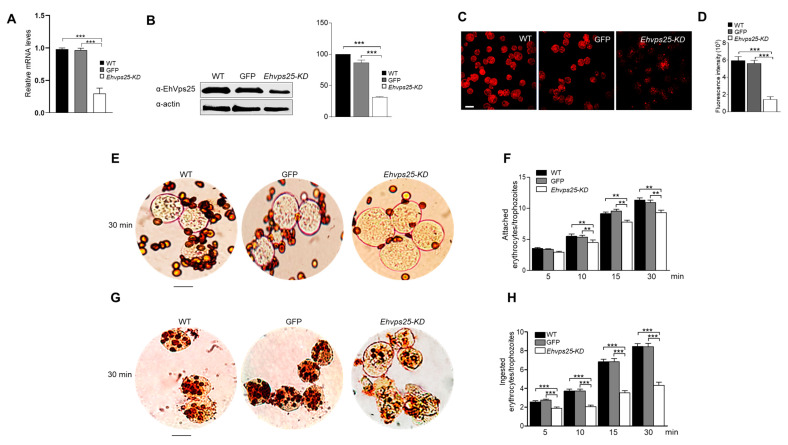
The phagocytic and adherence efficiency in trophozoites knocked down in the *Ehvps25* gene. (**A**) Relative mRNA levels obtained by RT-qPCR assay of trophozoites in basal conditions: WT, wild-type trophozoites; GFP, green fluorescent protein, used as a control for silencing of an unrelated gene in amoeba; *Ehvps25-KD*, knocked-down trophozoites. (**B**) Western blot assays. (**B**) Densitometry analysis of bands in (**A**) taking actin as a loading control. (**C**) Representative image of confocal microscopy of wild-type and *Ehvps25-KD* trophozoites (**D**) Fluorescence intensity measured by pixels. 30 fields in each sample were analyzed to obtain the graph. (**E**) Novikoff stain of wild type and *Ehvps25-KD* trophozoites. (**F**) Quantification of ingested erythrocytes. (**G**) Novikoff stain of wild type and *Ehvps25-KD* trophozoites. (**H**) Quantification of adhered erythrocytes. Data represent the mean and standard error of the erythrocytes number counted inside 100 trophozoites. ** *p* < 0.01, *** *p* < 0.001.

To study the role of ESCRT-II complex in phagocytosis, we generated trophozoites knocked-down in the *Ehvps25* gene using the double-stranded RNA (dsRNA) obtained from bacteria transfected with a plasmid containing the first 400 bp of the gene (in orientation 5′-3′) [42,43]. The trophozoites carrying the knocked-down gene expressed about 55% of the EhVps25 protein (Figure 8A,B), evaluated by western blot assays and using the green fluorescent protein (GFP) as an internal unrelated gene control [42]. Confocal microscopy images corroborated these results. The mutant trophozoites exhibited about 30% of the fluorescence intensity showed by the wild type and the internal control (Figure 8C,D). At all times tested, the knocked-down trophozoites presented almost 50% less rate of phagocytosis than the controls, and their efficiency to adhere RBCs was also affected (Figure 8E,F). However, adherence efficiency was not diminished as dramatically as the rate of phagocytosis (Figure 8G,H).

## 3. Discussion

The ESCRT complexes form a molecular machinery that, through subsequent and simultaneous protein interactions, facilitates vesicular trafficking and cargo transport from outside and inside the cell [3]. The deforming effect of ESCRT proteins on cellular membranes allows fusion and fission among the vesicles [44]. In addition to cell division, virus budding, and many other ESCRT driven events, in *E. histolytica*, this machinery participates in functions related to the capability of the parasite to produce damage to host cells, such as target cell adherence, phagocytosis, motility, and host cell destruction [24]. Hence, the study of ESCRT proteins is relevant for understanding the molecular events that occur in virulence-related functions of the trophozoites. In addition, *E. histolytica* presents high divergence in the proteins that constitute the conserved ESCRT machinery, producing divergent cellular and genomic features that are a challenging enigma from an evolutionary point of view. The knowledge of the molecules and their motifs that interact to perform these cellular events will give clues on the ancient protozoan’s evolutionary origin, particularly on the changes suffered by the ESCRT machinery.

Earlier, our group published the characterization of the proteins composing the ESCRT-I, and ESCRT-III complexes and the accessory proteins EhADH [24,45] and EhVps4 [18], all implicated in the virulence of the parasite. These studies have given us novel elements to understand their function and will help to find strategies to defeat amoebiasis, an infection that affects 3.55% of the world population and causes the death of between 50,000 and 75,000 people each year [46].

To construct a complete panorama of the ESCRT engine in trophozoites here, we presented the structural and functional characterization of the ESCRT-II proteins, emphasizing their interactions among them and with other ESCRT proteins and some others involved in phagocytosis. For our study, we followed a strategy based on three approaches: (i) By bioinformatics, we examined the structure and nature of the ESCRT-II proteins cloned and expressed in *E. coli*. Several structural features predicted that they are bona fide orthologues of the human and yeast ESCRT-II proteins and that they can interact with other proteins during vesicular trafficking in trophozoites. (ii) By specific antibodies, we detected and followed the movement of the ESCRT-II proteins and others participating in phagocytosis. We also explored the putative interaction of these molecules among them and with proteins of ESCRT-I and ESCRT-III complexes. (iii) Finally, we used the genetic approach to knock down one of the ESCRT-II genes (*Ehvps25*) and verified that phagocytosis was affected when this complex was altered.

Our results showed that *E. histolytica* possesses a complete canonical ESCRT-II complex, with differences between their human and yeast orthologues. While the three ESCRT-II proteins have the WH–WH domains involved in the interaction with nucleic acids and proteins, EhVps36 lacks the GLUE domain involved in tight interaction with Vps28 (ESCRT-I). The crystal structure of the GLUE domain missing this insertion reveals that it is a split PH domain with a non-canonical lipid pocket that binds PtdIns3P [3]. In the simultaneous and reinforcing interactions of GLUE domain with membranes, ESCRT-I and ubiquitin are critical for the ubiquitinated cargo progression from early to late endosomes [47]. However, through evolution, proteins have gained and lost different sequences that could modify their function. The similar position of EhVps22, EhVps25, and EhVps36 in the phylogenetic trees could suggest that these proteins evolved together and possibly together with other proteins of the ESCRT machinery to compensate for the losses that could affect the functions of the entire complex. This hypothesis needs to be strengthened with more bioinformatics and statistical data. In the case *of E. histolytica*, we have found that EhVps23 (ESCRT-I) binds ubiquitin [21], probably supplying the EhVps36 function. This interaction is not found very often in nature, but it has already been described for certain systems [5]. Other ESCRT proteins or motifs in the same protein could also carry out this task; for example, the N-terminal of the human Vps36 binds ubiquitin despite having no NZF domain [48], showing that proteins have other domains with an affinity for ubiquitin.

The ESCRT complexes have not been isolated; this could be explained because interactions among its proteins are continuous and short, making the isolation and purification of this machinery difficult. As an alternative for studying the ESCRT-III complex, we used the GUVs, an excellent system to in vitro prove and characterize the interactions among its proteins [14,16,49]. In the future, the ESCRT-II proteins can be further studied using GUVs. Meanwhile, by molecular docking we have simulated the association among the ESCRT-II proteins and the prediction suggests that EhVps23 and EhVps36 are able to bind with each other. Confocal immunofluorescence images also revealed proximity between these proteins. Although no association between Vps23 and Vps36 has been reported in humans and yeast, there are reports in wheat that they can associate with each other [50]. Thus, it is possible that this association also occurs in *E. histolytica*.

Laser confocal images of trophozoites also showed changes in the interactions and location of the ESCRT-II proteins under the stimulus of RBCs. In basal conditions, co-localization appeared in small areas, including in vesicles that seemed to be leaving the cell. Interestingly, after 2 min of contact with the RBCs, proteins also appeared in the phagocytic channel, the phagosomes, and the RBCs that were in the process of being ingested. The co-localization increased at 5 and 30 min, and again, we found both proteins interacting in vacuoles close to the ingested RBCs and close to adhered erythrocytes. The same events happened between EhVps25 and EhVps20; in the basal conditions, we observed poor co-localization, while during phagocytosis, the phagosomes and many internal vacuoles, as well as MBVs appeared with smaller vacuoles stained by both antibodies. The movement of the proteins inside the cell raises the possibility that they perform specific tasks for distinct functions at different cell regions and times. These interactions demonstrate the dynamism of ESCRT machinery during phagocytosis as it has been reported for other proteins involved in this phenomenon [51,52,53,54,55,56,57,58], reinforcing the assumption that ESCRT and other molecules work in concert to perform this vital nutrition task for the amoeba.

Mass spectrometry analysis corroborated the presence of the three ESCRT-II proteins in the pull-down samples. It revealed the presence of other ESCRT machinery proteins, probably working together: RabB GTPase, calmodulin, clathrin, and other molecules reported as involved in phagocytosis and virulence of the parasite appeared in the results of the analysis [30,31,32,33,34,35]. By the many proteins found in the mass spectrometry analysis, we postulate that the ESCRT-II complex could associate with other proteins to perform other processes in the trophozoites, such as cytokinesis; however, this speculation requires experimental strategies to be tested or discarded.

Finally, the evidence of the participation of the ESCRT-II complex in phagocytosis was given by the silencing experiments. Knocked-down trophozoites in the *Ehvps25* gene exhibited about 50% affectation in their rate of phagocytosis after 30 min in contact with RBCs. Adherence of the erythrocytes to the trophozoites was also significantly diminished, although diminishing was not as dramatic as phagocytosis rates. There are experimental results obtained from several other proteins involved in phagocytosis showing that when they are mutated, phagocytosis is affected [21,42,43,54], demonstrating the complexity of the event performed in a chain model in which several proteins are involved. Concatenated events occur with several proteins acting in concert to achieve the capture, transport, and digestion of the prey.

## 4. Materials and Methods

### 4.1. ESCRT-II Searching and Phylogenetic Analysis

Amino acids sequences corresponding to EhVps22 (EHI_131120), EhVps25 (EHI_137860), and EhVps36 (EHI_045320) proteins were obtained from the KEGG database (entries: K12188, K12189, and K12190) [59], and Amoeba DB [60] (https://amoebadb.org/amoeba/app; accessed on 26 November 2022) server; then, sequences were used for alignment with ClustalW [61]. The data were analyzed using MEGA 5.05 software. The bootstrapping was performed with 1000 replicates. Structural domains were identified using the SMART genomics server (http://smart.embl-heidelberg.de/; accessed on 26 November 2022) and the motif tool of KEGG (https://www.genome.jp/kegg/; accessed on 26 November 2022); the illustrations were done in S-Ilustrator for Biological Sequences (http://ibs.biocuckoo.org/online.php; accessed on 26 November 2022).

### 4.2. 3D Structure of EhVps22, EhVps25, EhVps36

Tertiary structures for EhVps22, EhVps25, EhVps36, and EhVps20 were predicted by the I-TASSER server [61,62] using homologous proteins from human ESCRT-II [63] (PDB: 3CUQ) or ESCRT-III [64] (PDB: 6ZH3) complexes as templates. Models were selected by the highest C-score, which were −0.82, 1.31, −1.09, and −2.90, respectively. Then, predicted structures were evaluated by Ramachandran plots at the Molprobity server [65] (http://molprobity.biochem.duke.edu/). The 3D model of EhVps23 was obtained as previously described by Galindo in 2021 [21]. Graphical representations were made with VMD version 1.9.3 software [66] 

### 4.3. Molecular Dynamics Simulation (MDS)

For molecular dynamics simulations, 3D structures were prepared with CHARMM-GUI server [67]. For the topology file, we used the CHARMM36 force field [68], and for system neutralization, the server added 48,388 water molecules, 278 sodium ions, and 282 chloride ions to EhVps22; 15,573 water molecules, 46 sodium ions, and 44 chlorides ions to EhVps25; 43,198 water molecules, 262 sodium ions, and 250 chloride ions to EhVps36; meanwhile, 161,894 water, 481 sodium, and 463 chloride molecules were added to the EhVps20 system. Solvation of the system was based on TIP3 water forming a cubic box with a margin of 10 Å to allow molecules to move during dynamics. Systems were minimized with 10,000 steps and equilibrated over 250,000 steps at NVT ensemble (constant volume and temperature) at 310 K through a nanosecond with protein atoms restrained. Generated files from equilibration were used to run 200-ns long MDS of each system under NPT ensemble maintaining constant temperature with Langevin dynamics and pressure at 1 bar with a Nosé–Hoover Langevin piston [69,70]. All proteins were considered soluble and without position restraints under periodic boundary conditions. We used a timestep of 2.0 fs and saved coordinates for all ps. All the MDS systems were run with NAMD 2.12 software [71] at Hybrid Cluster Xiuhcoatl (http://clusterhibrido.cinvestav.mx; accessed on 22 November 2022) of CINVESTAV-IPN, México.

### 4.4. Trajectory Analysis

MDS trajectory analysis, snapshots, and average structures were obtained with Carma software [72]. Root-mean square deviation (RMSD), the radius of gyration (Rg), and the root-mean square fluctuation (RMSF) were analyzed and plotted with Microsoft Excel software.

### 4.5. Protein–Protein Docking

The snapshots of EhVps23 [22], EhVps22, EhVps25, EhVps36, and EhVps20 for docking analysis were obtained using the clustering analysis in the last 50 ns of the MDS with the Carma software [72]. The protein–protein docking studies were calculated by employing different conformers with the ClusPro server [73,74]; the conformers with the highest cluster members and the lowest energy calculated in FireDock [75] were taken for analysis on the PDBsum server [76]. All 3D structure visualization was performed by VMD [66].

### 4.6. Culture of Trophozoites

Trophozoites of *E. histolytica*, clone A (strain HM1: IMSS) [77], were axenically cultured in TYI-S-33 medium at 37 °C and harvested in a logarithmic growth phase for all experiments [78].

### 4.7. Cloning of the E. histolytica vps22, vps25, and vps36 Genes (Ehvps22, Ehvps25, and Ehvps36)

The complete DNA sequences of *Ehvps22* (714 bp), *Ehvps25* (516 bp), and *Ehvps36* (708 bp) were PCR-amplified using the following specific primers: sense 5′CCGGTACCTACCCATACGATGTTCCAGATTACGCTATG.

TCAAAAAGAACAATTGATTTGTTATGTAG-3′ and antisense 5′-GGGGATCCTTAAAACAAGTTATATAATGATGTCCAATATGCT-3′, corresponding to *Ehvps22*; sense 5′CCGGTACCTACCCATACGATGTTCCAGATTACGCTATG.

ACATTTGGAATTCCTGAATTTGC-3′ and antisense 5′-GGGGATCCTTATTTCAA.

CCAAAAAATTCCATATTCTCCT-3′, corresponding to *Ehvps25*; and sense 5′CCGGTACCTACCCATACGATGTTCCAGATTACGCTATGTCTAATCAACCACAATATGGAATTAAATC-3′ and antisense 5′-GGGGATCCTTATTTGATGTATTGAAGGTATTGAGTATTAAAAAGA-3′, corresponding to *Ehvps36*. PCR assays were performed in a mixture containing 10 mM dNTPs, 100 ng of *E. histolytica* genomic DNA or cDNA as template, and 2.5 U of Taq DNA polymerase (Gibco). Amplification was carried out for 35 cycles (1 min at 94 °C, 30 s at 59 °C, and 40 s at 72 °C.) The sense oligonucleotides contain a SacI restriction site, while the antisense oligonucleotide includes a KpnI restriction site. The full-length genes were cloned in *pCold I DNA* plasmid, which conferred an N-terminal histidine tag.

### 4.8. PCR and RT-qPCR Assays

Genomic DNA (gDNA) and total RNA were isolated from trophozoites using the Wizard Genomic DNA Purification kit (Promega Corporation, Madison, WI, USA), and TRIzol reagent (Invitrogen, Waltham, MA, USA), respectively, according to the manufacturer’s recommendations. Complementary DNA (cDNA) was synthesized using oligo dT primers and the Superscript II reverse transcriptase (Invitrogen, Waltham, MA, USA). PCR amplifications were carried out using 50 ng of gDNA or cDNA as templates and specific primers. Reactions were performed in a 20 µL final volume, containing 1 µM each primer, 2 mM MgCl_2_, 200 µM dNTPs, 1X Taq buffer, and 1 U Taq DNA polymerase (Invitrogen). Cycling conditions included an initial denaturing step at 94 °C for 1 min, followed by 30 cycles of 94 °C for 1 min, 61 °C for 30 s, and 72 °C for 1 min, with a final extension step at 72 °C for 10 min. Products were separated by electrophoresis in 1% agarose gels and stained with ethidium bromide. As controls for PCR amplifications, instead DNA, nuclease-free water was used.

### 4.9. Expression and Purification of Recombinant Proteins and Generation of Anti-EhVps22, EhVps25, and EhVps36 Antibodies

*Escherichia coli* BL21 (pLysS) bacteria were separately transformed with the *pCold/Ehvp22*, *pCold/Ehvp25*, or *pCold/Ehvp36* constructs to produce recombinant proteins (rEhVps22, rEhVps25, and rEhVps36). The rEhVps22, rEhVps25, and rEhVps36 proteins were purified with cobalt beads in an imidazole gradient and used to subcutaneously and intramuscularly inoculate BALB/c mice (50 µg emulsified in Titer-Max Classic adjuvant, 1:1) (Sigma), Wistar rats (80 µg emulsified in Titer-Max Classic adjuvant, 1:1) (Sigma-Aldrich, St. Louis, MO, USA), and a New Zealand rabbit (150 µg emulsified in Titer-Max Classic adjuvant, 1:1) to generate α-EhVps22, α-EhVps25, and α-EhVps36 polyclonal antibodies, respectively. Two more doses with the same protein amount were injected at 15-day intervals, and animals were bled to obtain the immune serum. Pre-immune serum was also obtained before immunization.

### 4.10. Western Blot Experiments

Trophozoites lysates (40 µg), or purified rEhVps22, rEhVps25, and rEhVps36 were electrophoresed in 15% SDS-PAGE, transferred to nitrocellulose membranes and probed with mouse α-EhVps22 (1:1000), rat α-EhVps25 (1:2000), rabbit α-EhVps36 (1:1000), rat α-EhVps23 (1:500), rabbit α-EhVps20 (1:500), rabbit α-EhVps32 (1:500), and mouse α-actin (1:3000) antibodies. α-actin antibody was kindly donated by Dr. José Manuel Hernández (Cell Biology Department, CINVESTAV, México). Membranes were washed, incubated with α-mouse, α-rat, and α-rabbit HRP-labeled secondary antibodies (Sigma, 1: 10,000), and revealed with ECL Prime detection reagent (GE-Healthcare, Chicago, IL, USA), according to the manufacturer’s instructions.

### 4.11. Phagocytosis Assays

For phagocytosis assays, the trophozoites were incubated for 0, 2, 5, 15, and 30 min with RBCs (1:25) at 37 °C. At different times, trophozoites were prepared for immunofluorescence [45] and observed through the laser confocal microscope. Other samples were stained by the Novikoff technique [79], and adhered or ingested erythrocytes were counted in 100 randomly chosen trophozoites through a light microscope (Axiolab, Zeiss, Dublin, CA, USA).

### 4.12. Laser Confocal Microscopy Assays

Trophozoites were grown on coverslips, fixed with 4% paraformaldehyde at 37 °C for 1 h, permeabilized with 0.5% Triton X-100, and blocked with 10% fetal bovine serum in PBS. Then, cells were incubated at 4 °C overnight (ON) with either α-EhVps22 (1:100), α-EhVps25 (1:100), α-EhVps36 (1:100), α-EhVps23 (1:50), or α-EhVps20 (1:50) antibodies. After extensive washing, samples were incubated for 30 min at 37 °C with α-mouse pacific blue-labeled, α-rat TRITC-labeled, or α-rabbit FITC-labeled secondary antibodies (1:100). Nuclei were stained with 40′6-Diamidino-2- Phenylindole (DAPI). Fluorescence was preserved using VECTASHIELD antifade reagent (Vector), examined through a Carl Zeiss LMS 700 confocal microscope in laser sections of 0.5 µm, and processed with ZEN 2009 Light Edition Software (Zeiss, Dublin, CA, USA). We considered at least 25 confocal images using the ImageJ 1.45v software and JACoP plugin to evaluate the fluorescence intensity and co-localization between proteins.

### 4.13. Pull-Down Assays

Purified histidine-tagged EhVps22, EhVps25, and EhVps36 recombinant proteins were used. These were fixed to the cobalt resin by incubation for 3 h at 4 °C with gentle agitation. Subsequently, two washes were performed with an imidazole gradient. Then, a PBS wash was given. Resin-bound proteins were incubated with total trophozoite lysates for 4 h at 4 °C under gentle agitation. After this, three washes with elution buffer were performed. The samples were centrifuged, the supernatant was recovered, 2X sample buffer was added, and they were boiled for 5 min at 100°C. Subsequently, the samples were run by 12% SDS-PAGE and stained by Silver Stain (Bio-Rad Silver Stain Plus 161-0449, Hercules, CA, USA). The gel where the samples ran was cut and the samples were analyzed by mass spectrometry. 

### 4.14. Spectrometry Mass

The samples were analyzed on nano UPLC ACQUITY “M” Class coupled with a QToF Synapt G2-Si mass spectrometer (Waters Corporation, Milford, CT, USA) and analyzed by software Protein Lynx Global Server (PLGS) v3.0.3 (Waters Corporation, Milford, CT, USA). The data obtained were cured and selected as associations with each target protein if they had a reliability percentage greater than 95%. The reliability percentage is obtained during the peptide identification process (database search); the software ensures that each identified ion meets several physicochemical parameters, and the more models a given peptide “meets”, then its confidence increases. As negative control and to test the specificity of the associations, we performed the pull-down assay with the total amoeba extracts and the cobalt resin but without the recombinant protein of interest.

### 4.15. Silencing Assay

The knock-down (KD) of *Ehvps25* gene was performed using the bacterial expression of double-stranded RNA (dsRNA) and parasite soaking experiments as described by Solis and Guillén in 2008 [42]. Briefly, the first 400 bp from the 5′-end of the *Ehvps25* gene was PCR-amplified using the following primers: sense 5′-CCGAGCTCATGACATTTGGAATTCCTGAATTTGC-3′ and antisense 5′-GGGGTACCTTTCAATTGAAAATGATAAAGAATCTG-3′. Then, the amplicon was cloned into pJET1.2/blunt plasmid and cloned into *pL4440* plasmid, using the KpnI and BamHI restriction sites. PCR, restriction analysis, and DNA sequencing were performed to verify the resulting *pL4440-Ehvps25* plasmid. The competent RNase III-deficient *E. coli* strain HT115 was transformed with the *pL4440-Ehvps25* construct. Bacteria were grown at 37 °C in LB broth for plasmid construction or 2YT broth for dsRNA expression in the presence of ampicillin (100 mg/mL) and tetracycline (10 mg/mL). The expression of *Ehvps25* dsRNA was induced with 2-mM isopropyl β-D-1-thiogalactopyranoside ON at 16 °C. Then, the bacterial pellet was mixed with 1 M ammonium acetate and 10 mM EDTA, incubated with phenol:chloroform:isoamyl alcohol (25:24:1), and centrifuged. The supernatant was mixed with isopropanol and centrifuged, and the nucleic acid pellet was washed with 70% ethanol. DNase I (Invitrogen) and RNase A (Ambion, Austin, TX, USA) were added to eliminate ssRNA and dsDNA molecules. *Ehvps25*-dsRNA has rewashed with isopropanol and ethanol, analyzed by agarose gel electrophoresis, and the concentration was determined by spectrophotometry. Lastly, *Ehvps25*-dsRNA (50 µg/mL) purified molecules were added to trophozoites (3.0 × 104) in TYI-S-33 complete medium, and cultures were incubated at 37 °C for 24 and 48 h. At 24 h, silencing of the *Ehvps25* was analyzed by western blot assays and confocal microscopy. All subsequent experiments were done at 24 h. Trophozoites growing under standard conditions (without dsRNA), and another group with the *Gfp* gene knocked-down were used as controls wild type and unrelated gene, respectively.

### 4.16. Statistical Analysis

Statistical analyses were performed by *t*-Student test, using GraphPad Prism 5.0 software. The scores showing statistically significant differences are indicated with asterisks in the graphs. The corresponding p-values are indicated in figure legends.

## 5. Conclusions

**Figure 9:**
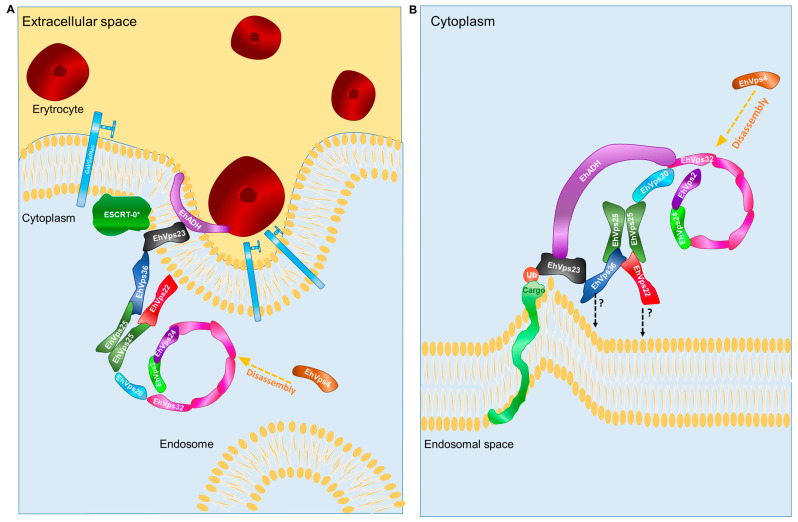
A working model that proposes the involvement of ESCRT-II in phagocytosis. (**A**) The erythrocytes stimulus triggers the recruitment to the plasma membrane of the ESCRT machinery. The EhADH, the Gal/GalNac lectin Kerp, and other molecules interact with the erythrocyte membrane. The asterisk indicates unidentified proteins performing functions of the ESCRT-0 complex could also participate in this binding. EhADH associates with EhVps23 (ESCRT-I) which interacts with EhVps36 and other members of the ESCRT-II complex. EhVps25 (ESCRT-II) recruits EhVps20 of the ESCRT-III complex. EhVps32 is then recruited by EhVps20 (ESCRT-III) and EhADH. These events promote the closure of the invagination formed by the adherence of the erythrocyte and the liberation of the nascent vesicle producing the erythrocyte containing endosome in the trophozoites cytoplasm. According to our results, the ESCRT machinery is involved starting from the adherence up to the MVB formation and also participates in secretion and motility. (**B**) Finally, EhVps32, EhVps20, EhVps24, and EhVps2 (ESCRT-III) polymerize, generating helical structures that produce the formation and closure of ILVs inside the MVBs. At the end of the process, EhVps4–ATPase disassembles the complex to restart the process.

All these results, together with others already published, demonstrate the central role of the ESCRT-II complex inside ESCRT machinery in phagocytosis and confirmed that all these proteins, together with EhADH, are actively participating since the contact through the introduction and conduction through the complex membranous system of the trophozoites to transport the cargoes molecules and the preys that can be RBCs, bacteria, and others (Figure 9).

## Data Availability

The data presented in this study are available in the article and Supplementary Materials.

## Supplementary Materials

The following supporting information can be downloaded at: https://www.mdpi.com/article/10.3390/ijms24065267/s1.
