# Supplementary material for: Dynamic Association of ESCRT-II Proteins with ESCRT-I and ESCRT-III Complexes during Phagocytosis of Entamoeba histolytica
Source: Int J Mol Sci. 2023 Mar 9;24(6):5267. doi: 10.3390/ijms24065267 (PMC10049522; doi:10.3390/ijms24065267)

Figure S1. Comparison of Vps22 amino acid sequences between *Homo sapiens*, *Saccharomyces cerevisiae* and *Entamoeba histolytica*.

```
Entamoeba histolytica      MSKRTIDLLCRQKEAMS-----TKGKEFCLVNQKERERQINLFKLKLEEFLQNNRYEITN
Homo sapiens               MHRRGVGAGAIAKKKLAEAKYKERGTVLAEDQLAQMSKQLDMFKTNLEEFASKHKQEIRK
Saccharomyces cerevisiae   MKQFGLAAF----DELKDGKYNDVNKTILEKQSVELRDQLMVFQERLVEFAKKHNSELQA
                           *  .  :        . :         .. :    :  :    *: :*: .* ** .::. *:

Entamoeba histolytica      DVNIQNMLYILCRKMGVEPLTTEKSYFN---IFFGSNAFYNELTVQIIEECYKSRESNGG
Homo sapiens               NPEFRVQFQDMCATIGVDPLASGKGFWS---EMLGVGDFYYELGVQIIEVCLALKHRNGG
Saccharomyces cerevisiae   SPEFRSKFMHMCSSIGIDPL----SLFDRDKHLFTVNDFYYEVCLKVIEICRQTKDMNGG
                           . ::.  :  :* .:*::**     . :.     ::  . ** *: :::** *    .  ***

Entamoeba histolytica      IIEINKLCELINKKR---KSIIRQNDIFKAINQAKCLGEGLKVVTIYQQSYVISVPFELA
Homo sapiens               LITLEELHQQVLKGRGKFAQDVSQDDLIRAIKKLKALGTGFGIIPVGGTYLIQSVPAELN
Saccharomyces cerevisiae   VISFQELEKVHFR-----KLNVGLDDLEKSIDMLKSL-ECFEIFQIRGKKFLRSVPNELT
                           :* :::* :    .         :  :*: .:*.  *.*    : :. :      : *** **

Entamoeba histolytica      DDQQVVLTLLNKKPYFLEDELLI-IGWSKERVKGTLVWLLNIGMLWVDDQFPLDGKTTRA
Homo sapiens               MDHTVVLQLAEKNGYVTVSEIKASLKWETERARQVLEHLLKEGLAWLDLQAPGEAH----
Saccharomyces cerevisiae   SDQTKILEICSILGYSSISLLKANLGWEAVRSKSALDEMVANGLLWIDYQGGAEAL----
                            *:  :* : .    *    . :    : *.  *  . .*  ::  *: *:* *    :.

Entamoeba histolytica      YWTSLY--NLF--------------
Homo sapiens               YWLPALFTDLYSQEITAEEAREALP
Saccharomyces cerevisiae   YWDPSWITRQL--------------
                           ** .
```

Figure S2. Comparison of Vps22 amino acid sequences between *Homo sapiens*, *Saccharomyces cerevisiae* and *Entamoeba histolytica*.

```
Entamoeba histolytica     --MTFGIPEFAKFPPFYTIQLVDKTKNQQLQLWSQLILKYCECIKKPIM----KQSEFNK
Homo sapiens              ---MSALPPVYSFPPLYTRQPNSLTRRQQISTWIDIISQYCKTKKIWYMSVDGTVINDNE
Saccharomyces cerevisiae  MAMSFEWPWQYRFPPFFTLQPNVDTRQKQLAAWCSLVLSFCRLHKQSSM----TVMEAQE
                                 *    ***::* *    *..:*:  * .:: .:*   *   *    .  : ::

Entamoeba histolytica     LP---------------IFHNEELHRTLSENGIELVKEFMVNNNKIIDLNKS--------
Homo sapiens              LDSGSTDNDDSKKISKNLFNNEDIQRSVSQVFIDEIWSQMTKEGKCLPIDQSGRRSSNTT
Saccharomyces cerevisiae  SP---------------LFNNVKLQRKLPVESIQIVLEELRKKGNLEWLDKS--------
                                           :*:* .::*.:.   *: : . : ::.:   :::*

Entamoeba histolytica     -SKLILLYKPLREWGKELYEYGNSKGLIGQSDTFFSIENDKESV---FYQMDDELLIEGL
Homo sapiens              TTRYFILWKSLDSWASLILQWFEDSGKLNQVITLYELSEGDETVNWEFHRMPESLLYYCL
Saccharomyces cerevisiae  KSSFLIMWRRPEEWGKLIYQWVSRSGQNNSVFTLYELTNGEDTEDEEFHGLDEATLLRAL
                           :  ::::.   .*.. : ::  . .*  ..  *::.: :..::    *: : :  *   *

Entamoeba histolytica     NSIKEQGKMKLVQHEGEY--GIFWLK
Homo sapiens              KPLCDRNRATMLKDENDKVIAIKVV-
Saccharomyces cerevisiae  QALQQEHKAEIITVSDGR--GVKFF-
                          :.: :  .  ::  ..    .:  .
```

Figure S3. Comparison of Vps36 amino acid sequences between *Homo sapiens*, *Saccharomyces cerevisiae* and *Entamoeba histolytica*.

```
Entamoeba histolytica      ------MDRFVWTSGLLEINETLVIQQRGVRIYDGEEKI-KFDAGTLLLSTHRLIWRDQ-
Homo sapiens               ------------------------------------------------------------
Saccharomyces cerevisiae   MEYWHYVETTSSGQPLLREGEKDIFIDQSVGLYHGKSKILQRQRGRIFLTSQRIIYIDDA

Entamoeba histolytica      KNHECCMAILLSQIVFIEEQAAGIGKSAKIVVHLHPAP----------------------
Homo sapiens               ------------------------------------------------------------
Saccharomyces cerevisiae   KPTQNSLGLELDDLAYVNYSSGFLTRSPRLILFFKDPSSKDELGKSAETASADVVSTWVC

Entamoeba histolytica      ----------------------------------------------PNKEP---------
Homo sapiens               ----------------------------------------------MSNQP---------
Saccharomyces cerevisiae   PICMVSNETQGEFTKDTLPTPICINCGVPADYELTKSSINCSNAIDPNANPQNQFGVNSE
                                                                          . :*

Entamoeba histolytica      ------------------------------------------------------------
Homo sapiens               ------------------------------------------------------------
Saccharomyces cerevisiae   NICPACTFANHPQIGNCEICGHRLPNASKVRSKLNRLNFHDSRVHIELEKNSLARNKSSH

Entamoeba histolytica      -----GPFQSSKNSYIKLSFKEHGQIEFYRR--------LSEEMTQRRWENMPVSQSLQT
Homo sapiens               ------------------------------------------------------------
Saccharomyces cerevisiae   SALSSSSSTGSSTEFVQLSFRKSDGVLFSQATERALENILTEKNKHIFNQNVVSVNGVDM

Entamoeba histolytica      NRGPQP-----------GRIRAVGIVGIERKLEEKRKETDKNISEAFEDLSKLMIKAKEM
Homo sapiens               ---------------------QYGIKSYIEAKEKDQNDIDNLFSNAFESLDTVIQYWDQL
Saccharomyces cerevisiae   RKGASSHEYNNEVPFIETKLSRIGISSLEKSRENQLLNNDILFNNALTDLNKLMSLATSI
                                                  ** .     *:.  : *  :.:*: .*..::    .:

Entamoeba histolytica      VELSKSIANKIKDKQGDITEDETIRFKSYLLSMGIANPVT--------RETYGSGTQYHM
Homo sapiens               DKVATKFL-----KESNM-EKENHEYQSLITYLDITEPVT--------KKLFGNN--YEE
Saccharomyces cerevisiae   ERLYKNSNITMKTKTLNL-QDEST----------VNEPKTRRPLLILDREKFLNKELFLD
                             : ..       *  :: :.*.           : :* *        .: : .   :

Entamoeba histolytica      QLAKQLAGILQVPLEERGG-----IMSLTEVYCLVNRAR--GMELLSPEDLVNACKMLEA
Homo sapiens               ALMDEIDEFCYLYFQKKKE----MAIMLSDLYAIYNRAR--GI-LISPKELRNVCELLND
Saccharomyces cerevisiae   EIARELYEFTLSEFKDLNSDTNYMIITLVDLYAMYNKSMRIGTGLISPMEMREACERFEH
                            :  ::  :    ::.         : * ::*.: *.:   *  *:** :: :.*: ::

Entamoeba histolytica      LKLP-LRLRVFDSGVMVIELQSHKE--EEMVASA-----LETVSEKGSLTSEEFAKLVGM
Homo sapiens               KGKY-VGLMNVNKS-LIIHERDFTL--EKFQNTI-----LDALKSRQKISSFTIAQHYGI
Saccharomyces cerevisiae   LGLNELKLVKVNKRILCVTSEKFDVVKEKLVDLIGDNPGSDLLRLTQILSSNNSKSNWTL
                                : *  .:.  : :   ..    *::          : :     ::*    .   :

Entamoeba histolytica      SVLLAKERLLLAEKMGHLCRDDSVEGLRFYPNLFMTQS-----
Homo sapiens               QYAIVQTLMDQLEQNELICRDESEEGIMYYENLFNTQYLQYIK
Saccharomyces cerevisiae   GILM--EVLQNCVDEGDLLIDKQLSGIYYYKNSYWPSHI----
                              :    :    .   :  *.. .*: :* * : ..
```

Figure S4. EhVps22, EhVps25, and EhVps36 proteins conserve the WH domains.

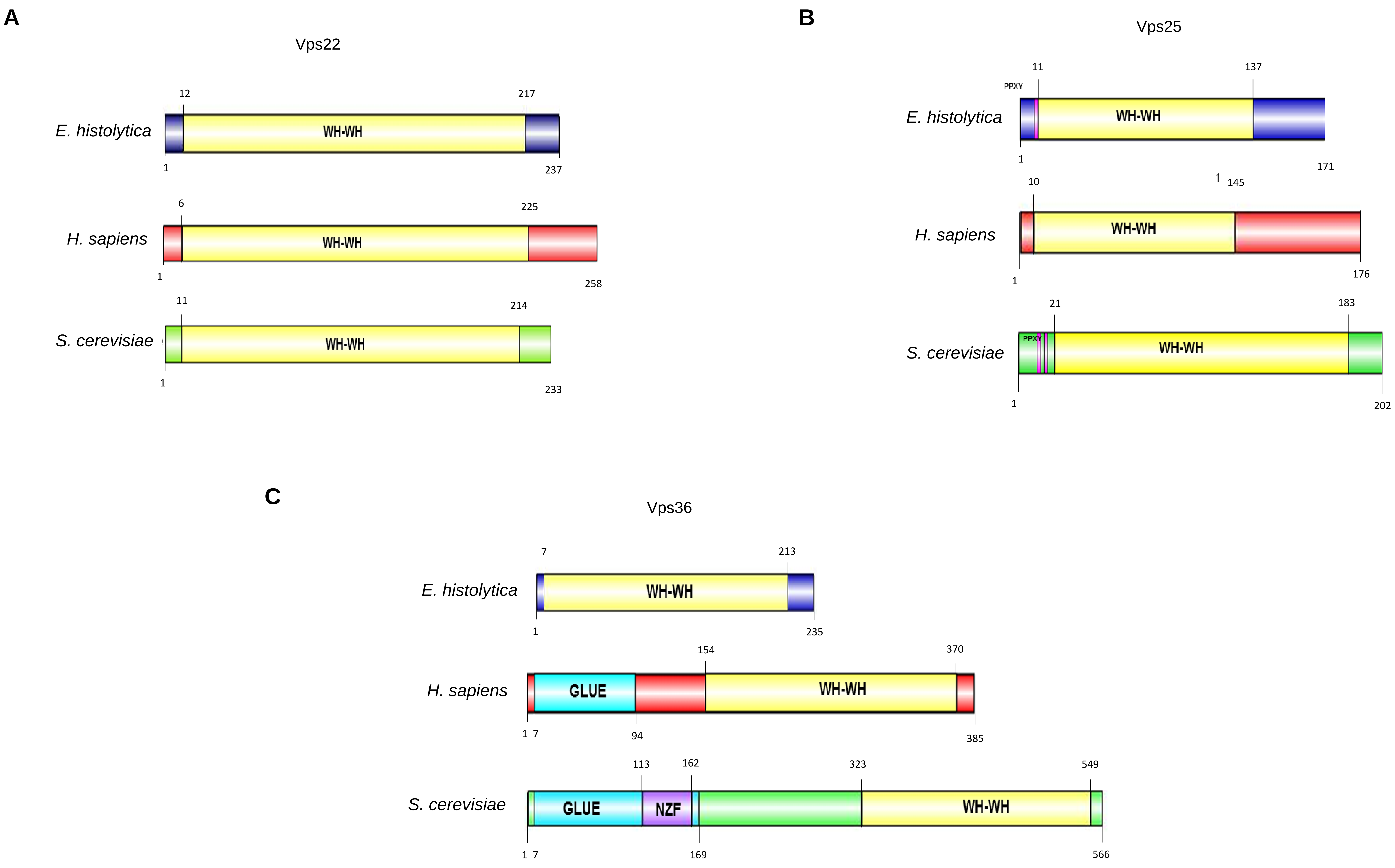

Figure S5. Predicted model of EhVps22, EhVps25, and EhVps36 MDS results

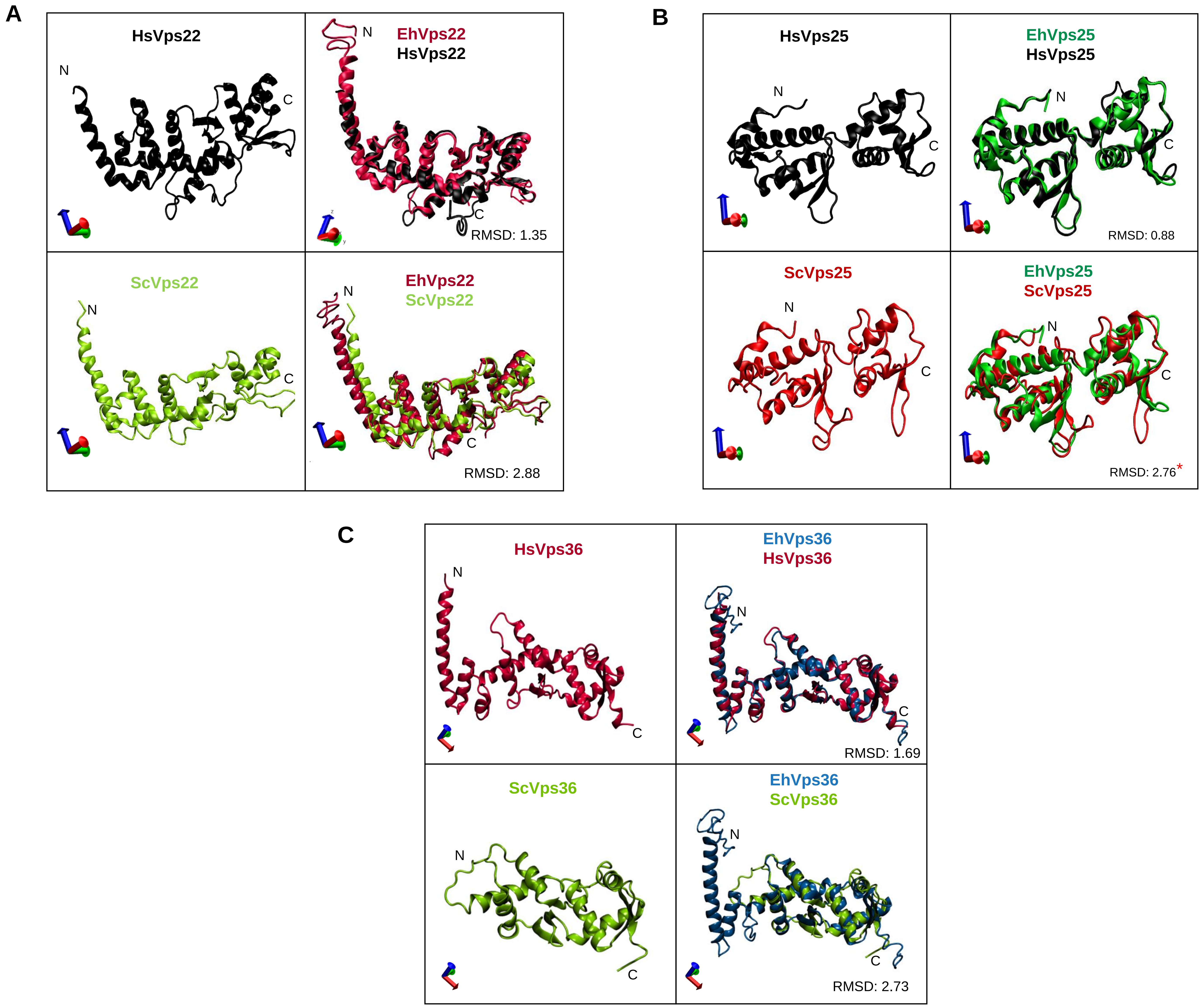

Supplement: Supplementary file 1 [file ijms-24-05267-s001.zip › ijms-2144858-supplementary.pdf]
